# Covid-19 Dynamic Monitoring and Real-Time Spatio-Temporal Forecasting

**DOI:** 10.3389/fpubh.2021.641253

**Published:** 2021-04-08

**Authors:** Cecilia Cordeiro da Silva, Clarisse Lins de Lima, Ana Clara Gomes da Silva, Eduardo Luiz Silva, Gabriel Souza Marques, Lucas Job Brito de Araújo, Luiz Antônio Albuquerque Júnior, Samuel Barbosa Jatobá de Souza, Maíra Araújo de Santana, Juliana Carneiro Gomes, Valter Augusto de Freitas Barbosa, Anwar Musah, Patty Kostkova, Wellington Pinheiro dos Santos, Abel Guilhermino da Silva Filho

**Affiliations:** ^1^Center for Informatics, Federal University of Pernambuco, Recife, Brazil; ^2^Nucleus for Computer Engineering, Polytechnique School of the University of Pernambuco, Recife, Brazil; ^3^Department of Biomedical Engineering, Federal University of Pernambuco, Recife, Brazil; ^4^Academic Unit of Serra Talhada, Rural Federal University of Pernambuco, Serra Talhada, Brazil; ^5^Institute for Risk and Disaster Reduction, University College London, London, United Kingdom

**Keywords:** COVID-19, SARS-CoV-2, Covid-19 pandemics forecasting, spatio-temporal analysis, spatio-temporal forecasting, digital epidemiology

## Abstract

**Background:** Periodically, humanity is often faced with new and emerging viruses that can be a significant global threat. It has already been over a century post—the Spanish Flu pandemic, and we are witnessing a new type of coronavirus, the SARS-CoV-2, which is responsible for Covid-19. It emerged from the city of Wuhan (China) in December 2019, and within a few months, the virus propagated itself globally now resulting more than 50 million cases with over 1 million deaths. The high infection rates coupled with dynamic population movement demands for tools, especially within a Brazilian context, that will support health managers to develop policies for controlling and combating the new virus.

**Methods:** In this work, we propose a tool for real-time spatio-temporal analysis using a machine learning approach. The COVID-SGIS system brings together routinely collected health data on Covid-19 distributed across public health systems in Brazil, as well as taking to under consideration the geographic and time-dependent features of Covid-19 so as to make spatio-temporal predictions. The data are sub-divided by federative unit and municipality. In our case study, we made spatio-temporal predictions of the distribution of cases and deaths in Brazil and in each federative unit. Four regression methods were investigated: linear regression, support vector machines (polynomial kernels and RBF), multilayer perceptrons, and random forests. We use the percentage RMSE and the correlation coefficient as quality metrics.

**Results:** For qualitative evaluation, we made spatio-temporal predictions for the period from 25 to 27 May 2020. Considering qualitatively and quantitatively the case of the State of Pernambuco and Brazil as a whole, linear regression presented the best prediction results (thematic maps with good data distribution, correlation coefficient >0.99 and RMSE (%) <4% for Pernambuco and around 5% for Brazil) with low training time: [0.00; 0.04 ms], CI 95%.

**Conclusion:** Spatio-temporal analysis provided a broader assessment of those in the regions where the accumulated confirmed cases of Covid-19 were concentrated. It was possible to differentiate in the thematic maps the regions with the highest concentration of cases from the regions with low concentration and regions in the transition range. This approach is fundamental to support health managers and epidemiologists to elaborate policies and plans to control the Covid-19 pandemics.

## 1. Introduction

In December 2019, in the city of Wuhan, one of the most populated cities in China, there was an atypical pneumonia outbreak attributed to the new coronavirus, SARS-CoV-2. The disease was called Coronavirus Disease 2019, or Covid-19. This infectious disease spread rapidly across China, coinciding with the Spring Festival on January 25. During this festival, massive population movements across the country contributed to the Covid-19 epidemic in China (1). The World Health Organization (WHO) rapid declared Covid-19 a Public Health Emergency of International Interest (PHEIC) on January 30, 2020 (2). The WHO classified Covid-19 as a pandemic on March 11. Since then, it has been a major public health challenge worldwide. The International Health Regulatory Committee reaffirmed the declaration of January 30, as Covid-19 continues to constitute a PHEIC (3). According to the WHO, between December 2019 and May 2020, more than 4.7 million people were infected in 216 countries.

In a globalized context, where all nations are culturally, physically and economically interconnected, means of transport contribute to the rapid spread of infectious diseases. In this way, Covid-19 spread rapidly across China and Western Europe. In a short time, the Covid-19 epidemic had become a pandemic, worrying national health authorities and the World Health Organization. Governments, universities, public and private research institutes and various social actors began to invest in the search for new methods of diagnosis of Covid-19 and detection of the new coronavirus, SARS-CoV-2, in addition to treatments, specific drugs and vaccines to combat Covid-19. At the same time, measures of social isolation and confinement are taken by national and local governments, while false news goes viral on various social media, aiming to disseminate ineffective and dangerous early treatments, drugs used outside the context for which they were conceived, and wrong scientific tests. Often this false information was released with the support of political leaders and famous people from important nations, such as the United States and Brazil.

Against epidemics that spread with high rates of infection and whose vector is the human being itself, measures to contain epidemics, such as social distance, confinement, inter and intranational border control applied by national states, in addition to the use of facial masks, use of non-pharmacological measures and individual hygienic measures, such as regular hand hygiene and disinfection, are extremely necessary, as demonstrated in a study performed in India by Samui et al. (4). The public and private media have played a fundamental role in this historic moment to encourage the population to follow health rules and give up individual and collective freedoms in favor of a greater good, as demonstrated by Khajanchi et al. (5). Right now, most countries in the world are experiencing the emergence of a second wave of contagion, largely due to the emergence of new strains of SARS-CoV-2, which are more contagious and, at times, more deadly. The measures of social detachment and confinement, previously loosened, are now being resumed with more rigor. Although there are already losses in individual and collective consciences that have gradually reduced the effectiveness of measures of social distance and confinement, mainly by adolescents and young adults, the measures are still necessary until the combination of two factors is present: mass vaccination of the majority of the population and the creation and popularization of specific treatments and drugs to treat moderate and severe cases of Covid-19, and consequently, reduce treatment time, bed occupancy rate, and mortality.

In order to seek solutions for the control of the disease, several studies have been done, such as the development of GIS (Geographic Information System) platforms for monitoring the number of confirmed cases as well as deaths and recoveries of Covid-19 in regions of China, USA, Australia, Brazil, and Canada (6–14). Other studies include the analysis of the Covid-19 virus genome, like the researches carried out by Randhawa et al. (15), in order to analyze and understand the virus. Some studies help in our understanding of the diagnosis of the disease using relatively novel methods like artificial intelligence and machine learning, as shown by Xie et al. (16), Feng et al. (17), Jin et al. (18), Meng et al. (19), and Barbosa et al. (20).

Over the days, the number of cases has increased, as well as the number of deaths, and in order to prevent the collapse of health systems and a greater control of the evolution of the disease, many researchers from around the world have begun to study ways to predict the number of Covid-19 cases using Machine Learning methods as done by Gupta and Pal (21), Pandey et al. (22), and Tomar and Gupta (23). These studied the distribution of cases and made predictions for India. Sarkar et al. (24) and Khajanchi and Sarkar (25) propose novel epidemiological compartment models for Covid-19 infected and deaths forecasting, using data for India as case studies. Regarding statistical learning models, Ndaïrou et al. (26), similarly to Sarkar et al. (24) and Khajanchi and Sarkar (25), built a novel epidemiological compartment model for Covid-19, but applied their model to data from Wuhan, China. In the same vein, Yang et al. (1) made case predictions for China, in addition to Jiang and Schotten (27), applying state-of-the-art machine learning methods for not only modeling the burden of Covid-19 with artificial neural networks, but also going a step further toward model assessment and performance evaluation.

## 2. Related Works

After the outbreak in the city of Wuhan, China, several researchers began to study the coronavirus in order to analyze and understand the behavior of the disease and even predict the evolution of cases. In this way, these studies could guide government agencies in promoting the health and quality of life of the population during the pandemics.

Through a system of ordinary differential equations, the disease is contextualized through social parameters to understand how the spread works and how it is possible to control the epidemics that affect society and thereby create preventive measures. Examples of this type of model are the modified SEIR models proposed by Yang et al. (1) as well as the SEIR (Susceptible, Exposed, Infectious, Recovered) model with age-structured quarantine class with the two types of control measures used to analyze the effects of policy control for the coronavirus epidemic in Brazil (28), and the SEIRQ (Susceptible, Exposed, Infectious, Recovered, Quarantine) model with age structure, proposed by Gondim and Machado (29). This model aims to analyze optimal quarantine strategies in order to help in decision-making through health managers.

Several efforts to aid Covid-19 screening and monitoring can be perused in the works of Dong et al. (6). In this work, Dong et al. (6) created an online interactive panel to visualize Covid-19 infected cases and deaths in real time, providing researchers, health authorities, and the general public a tool to track cases as the disease progresses. Due to the rapid development of the coronavirus, the need to classify infected patients and analyze which individuals were more vulnerable to the disease also grew, Therefore, Xie et al. (16) proposed a model of clinical prediction for patient mortality based on multivariable logistic regression, to improve the use of limited healthcare resources and calculate the patient's survival rate. Furthermore, in order to aid the diagnosis, Feng et al. (17) developed the online calculator S-COVID-19-P based on Lasso regression, for early identification of suspected Covid-19 pneumonia in the admission of adult patients with fever. Jin et al. (18) proposed a system based on deep learning for the rapid diagnosis of Covid-19 with precision comparable to experienced radiologists, and can accurately classify pneumonia, CAP (Community-Acquired Pneumonia), influenza A and B and Covid-19. They used LASSO to find the 12 most discriminating characteristics in the distinction between Covid-19 and other pneumonias. Gomes et al. (30) proposed a system to support the diagnosis of Covid-19 by analyzing chest X-ray images, capable of differentiating Covid-19 from bacterial and viral pneumonias using texture-based image representation and classification by Random Forests. Different from other more complex Covid-19 x-ray feature extraction approaches (31–42), Gomes et al. (30) avoided deep learning based solutions and adopted texture and shape features to provide the users a low-cost computational web-based computational environment able to deal with several simultaneous users without overcharging network resources.

In order to find a new way to perform early, efficient, and accurate control and screening of suspected individuals, Meng et al. (19) created the Covid-19 Diagnostic Aid APP to calculate the probability of infection through simple and easy laboratory test results. Screening a large number of suspicious people could optimize the diagnostic process and save medical resources. Barbosa et al. (20) considered the fact that, in many regions of the world, RNA testing is not always available due to the scarcity of inputs, created HegIA, an intelligent system based on Bayes Networks and Random Forests to aid at the diagnosis of Covid-19 based on blood tests from 24 blood tests. The performance is close to RT-PCR (Reverse Transcription Polymerase Chain Reaction) for symptomatic individuals, though coronavirus RNA is not searched (20). HegIA is a fully functional system, available for free use, to provide low-cost rapid testing.

Regarding statistical epidemiological models, Sarkar et al. (24) propose a mathematical model to monitor the dynamics of six compartments: Susceptible (S), Asymptomatic (A), Recovered (R), Infected (I), Isolated Infected (Iq), and Quarantined Susceptible (Sq), collectively expressed SARIIqSq. The authors applied their proposal to real data on the COVID-19 pandemic in India. Starting from the date of first COVID-19 case reported in India, the authors have simulated the SARIIqSq model for 260 days for each states and for whole India to study the dynamics of the SARS-CoV-2 disease. They statistically confirmed that a reduction in the contact rate between uninfected and infected individuals by quarantined the susceptible individuals can effectively reduce the basic reproduction number. They also demonstrate that the elimination of ongoing SARS-CoV-2 pandemic is possible by combining the restrictive social distancing and contact tracing. However, the authors also emphasize the uncertainty of accessible authentic data, specially concerning to the accurate baseline number of infected individuals due to subnotifications, which may guide to equivocal outcomes and inappropriate predictions by orders of size.

Ndaïrou et al. (26) propose a novel epidemiological compartment model that takes into account the super-spreading phenomenon of some individuals. They consider a fatality compartment, related to death due to the virus infection. The constant total population size N is subdivided into eight epidemiological classes: Susceptible class (S), Exposed class (E), Symptomatic and Infectious class (I), Super-Spreaders class (P), Infectious but Asymptomatic class (A), Hospitalized (H), Recovery class (R), and Fatality class (F). This model reached a reasonably good approximation of the reality of the Wuhan outbreak, predicting a diminishing on the daily number of confirmed cases of the disease. The model also fits well the real data of daily confirmed deaths. The model can be considered useful for other realities than Wuhan, China, since the amount of hospitalized individuals is relevant as an estimate of the Intensive Care Units (ICU) needed.

The basic reproduction number *R*_0_ is one of the most crucial quantities in infectious diseases, since *R*_0_ measures how contagious an infectious disease is (43). For *R*_0_ < 1, the disease is expected to stop spreading. Nevertheless, for *R*_0_ = 1, an infected individual can infect on an average 1 person, i.e., the spread of the disease is stable. The infectious disease can spread and become an epidemics in case *R*_0_ > 1 (43).

Khajanchi and Sarkar (25) developed a new compartmental model to explain the transmission dynamics of Covid-19. They calibrated their model with daily Covid-19 data for four Indian states: Jharkhand, Gujarat, Andhra Pradesh, and Chandigarh. They studied the feasible equilibria of the proposed model and their stability with respect to the basic reproduction number *R*_0_. The disease-free equilibrium becomes stable and the endemic equilibrium becomes unstable when the recovery rate of infected individuals increases, but if the disease transmission rate remains higher, then the endemic equilibrium always remains stable. The proposed model obtained *R*_0_ > 1 for all studied Indian states, suggesting a significant outbreak. The model is able to provide short-time Covid-19 forecasting as well.

Samui et al. (4) proposed a deterministic ordinary differential equation model able to represent the overall dynamics of SARS-CoV-2. They stratified the total human population into four compartments: susceptible individuals (uninfected), asymptomatic individuals (pauci-symptomatic or clinically undetected), reported symptomatic infected individuals (symptomatic infectious individuals are reported by the public heath service) and unreported symptomatic infected individuals (clinically ill but not reported) to formulate the SAIU [susceptible or uninfected (S), asymptomatic (A), reported symptomatic infectious (I), unreported symptomatic infectious (U)] model. This model is based on the assumption that the reported infected individuals will no-longer associate into the infections as they are isolated and move to the hospital or Intensive Care Units (ICU). Thus, only infectious individuals belonging to I(t) or U(t) spread or transmit the diseases. The authors designed the SAIU model to study the transmission dynamics of COVID-19 based on the accessible data for India during the time period January 30, 2020 to April 30, 2020. Based on the estimated data, the SAIU model predicts the outbreak of COVID-19 and computes the basic reproduction number *R*_0_. The authors assessed the sensitivity indices of the basic reproductive number *R*_0_, given that *R*_0_ expresses the initial disease transmission and the sensitivity indices describes the relative importance of various parameters in coronavirus transmission. The SAIU model showed the persistence of diseases for *R*_0_ > 1. The endemic equilibrium point E*, for this study, was locally asymptotically stable for *R*_0_ > 1.

Khajanchi et al. (5) extended the classical deterministic susceptible-exposed-infectious-removed (SEIR) compartmental model refined by introducing contact tracing-hospitalization strategies to study the epidemiological properties of Covid-19. They calibrated their mathematical model using data of confirmed cases in India and estimated the basic reproduction number for the disease transmission. The authors have their calibrated epidemic model for the short term prediction in the four provinces and the Republic of India. The simulation of the calibrated model was able to capture the increasing growth patterns for three different provinces, namely Delhi, Maharashtra, West Bengal, and the Republic of India, whereas in case of the province Kerala, the model fitting is not good compared to other states and overall India. Model simulation and prediction suggest that Covid-19 has a potential to exhibit oscillatory but controllable dynamics in the near future by maintaining social distancing and effectiveness of home isolation and hospitalization. The proposed model forecasts that isolation or hospitalization of the symptomatic population, under stringent hygiene safeguards and social distancing, is considerably effective. Finally, Khajanchi et al. (5) give evidences that the size and duration of an epidemic can be considerably affected by timely implementation of the hospitalization or isolation programme.

The classic mathematical models of epidemiological prediction are quite useful, but deterministic, demonstrating only the average behavior of the epidemic, which makes it difficult to quantify uncertainty. Wang et al. (44) proposed an analysis of the spatial structure and dynamics of the spread of Covid-19, providing a spatio-temporal prediction of the Covid-19 outbreak in the United States. Kapoor et al. (45) investigated large-scale spatio-temporal prediction using neural network graphs and human mobility data in US counties. Through this method and space-time information, the model learns the epidemiological dynamics. Tomar and Gupta (23) proposed a space-time approach to control and monitor Covid-19 using LSTM (Long Short-Term Memory) neural networks and adjusting curves to predict chaos. Ren et al. (46) used Ecological Niche Models (ENM) to gather epidemiological and socioeconomic data, aiming to accurately predict the risk areas for Covid-19 infection. Yesilkanat (47) made a study with space-time approach for 190 countries in the world and compared it with the numbers of real cases of the disease using the Random Forest method. Also using a space-time approach, Pourghasemi et al. (48) did a risk mapping, change detection and trend analysis of the Covid-19 spread in Iran using regression and machine learning. Roy et al. (49) developed a short-term prediction model for the new Coronavirus using canonical ARIMA (Autoregressive Integrated Moving Average) and disease risk analysis done using weighted overlap analysis in geographic information systems.

## 3. Materials and Methods

### 3.1. Proposed Method

**Figure 1:**
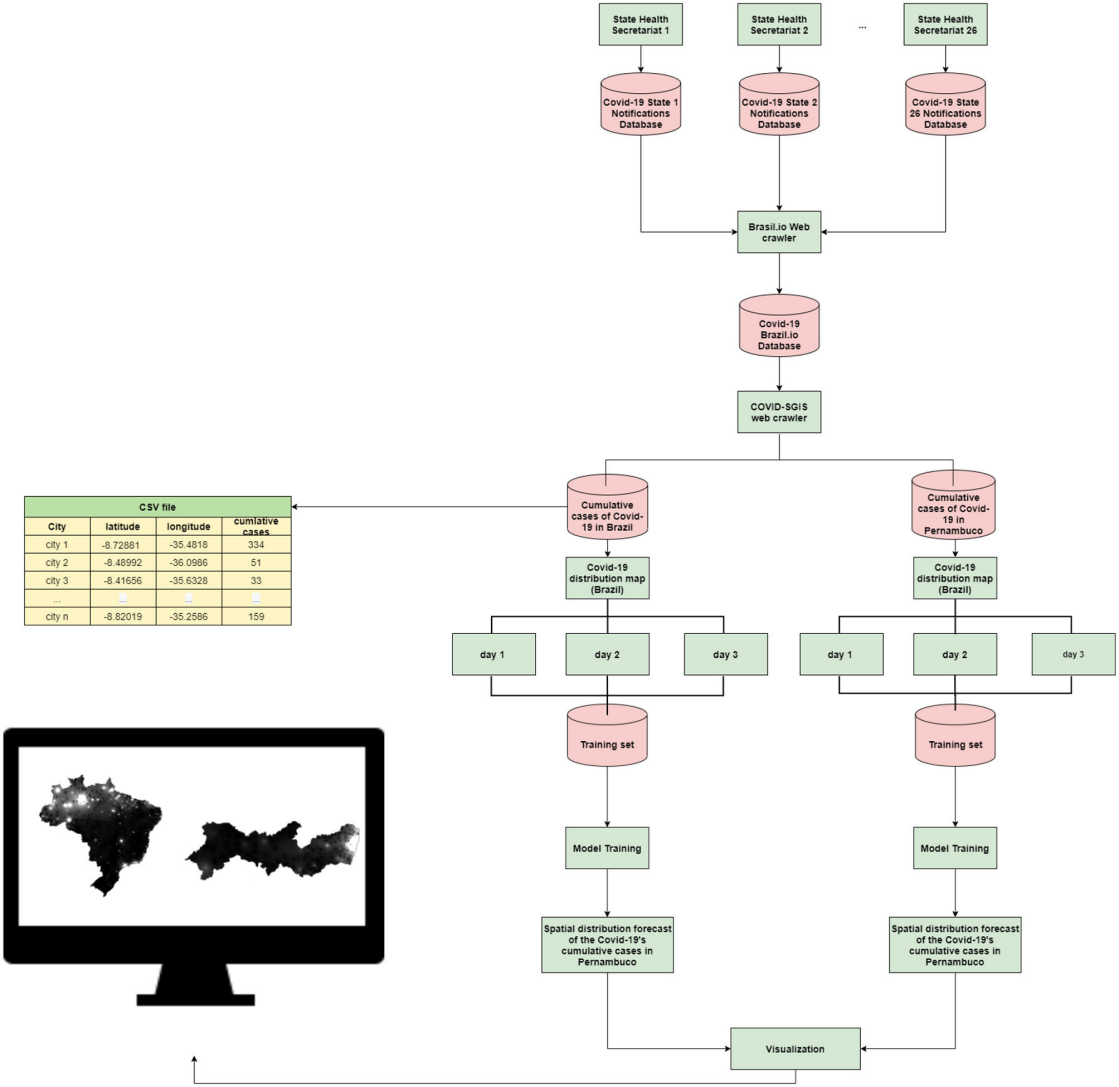
Proposed method: each of the 26 state health departments is responsible for issuing Covid-19 epidemiological bulletins. This information is available on the Brasil.io portal. Our software, COVID-SGIS, collects confirmed cases of Covid-19 for the municipalities of Pernambuco and for the municipalities of Brazil on a daily basis. A CSV file is organized with information on latitude, longitude and accumulated cases of the disease. Then, case distribution maps for both territories are generated separately. The prediction sets are assembled so that the maps of 3 consecutive days are used for the projection of 3 days ahead. The user can view the 3-day projection maps for Brazil and Pernambuco.

In this work, we proposed a system to forecast the spatio-temporal distribution of Covid-19 in Brazil, and in the State of Pernambuco (which is a Brazilian state). The system operates as follows: Each municipality in Brazil and in Pernambuco, feeds a database of Covid-19 notifications. All this information is obtained through the Brasil.io portal (https://brasil.io/dataset/covid19/case/). Then, our software, the COVID-SGIS, collects daily information of Covid-19 cases for both territories, separately. Then, we calculate the disease's cumulative number for each municipality for the State of Pernambuco and for the whole country as well. This information is stored in a comma-separated file (.csv), in which each file contains information about the accumulated number of cases and the municipality coordinates. Thereafter, the databases generated in the previous step are sent to an interpolation module to assemble the training datasets. In this module, the number of cases is distributed in a inhomogeneous grid by using latitude and longitude of the corresponding municipality centroid. Then, the interpolation module generates a regular grid. Each point of this regular grid is calculated using the Inverse Distance Weighting interpolation (IDW) method. This process is followed to estimate the distribution of both infected cases and deaths. Then generate spatial distribution maps for each notification day. Thus, the distribution maps are organized so that maps of 3-consecutive days are used to forecast the distribution map of the following day. With the models created, 3-day spatial forecasts are generated. Finally, the software presents the user with the prediction maps of the accumulated cases of Covid-19 for Brazil and for Pernambuco. Figure 1 graphically illustrates our proposal.

### 3.2. Areas Under Study

**Figure 2:**
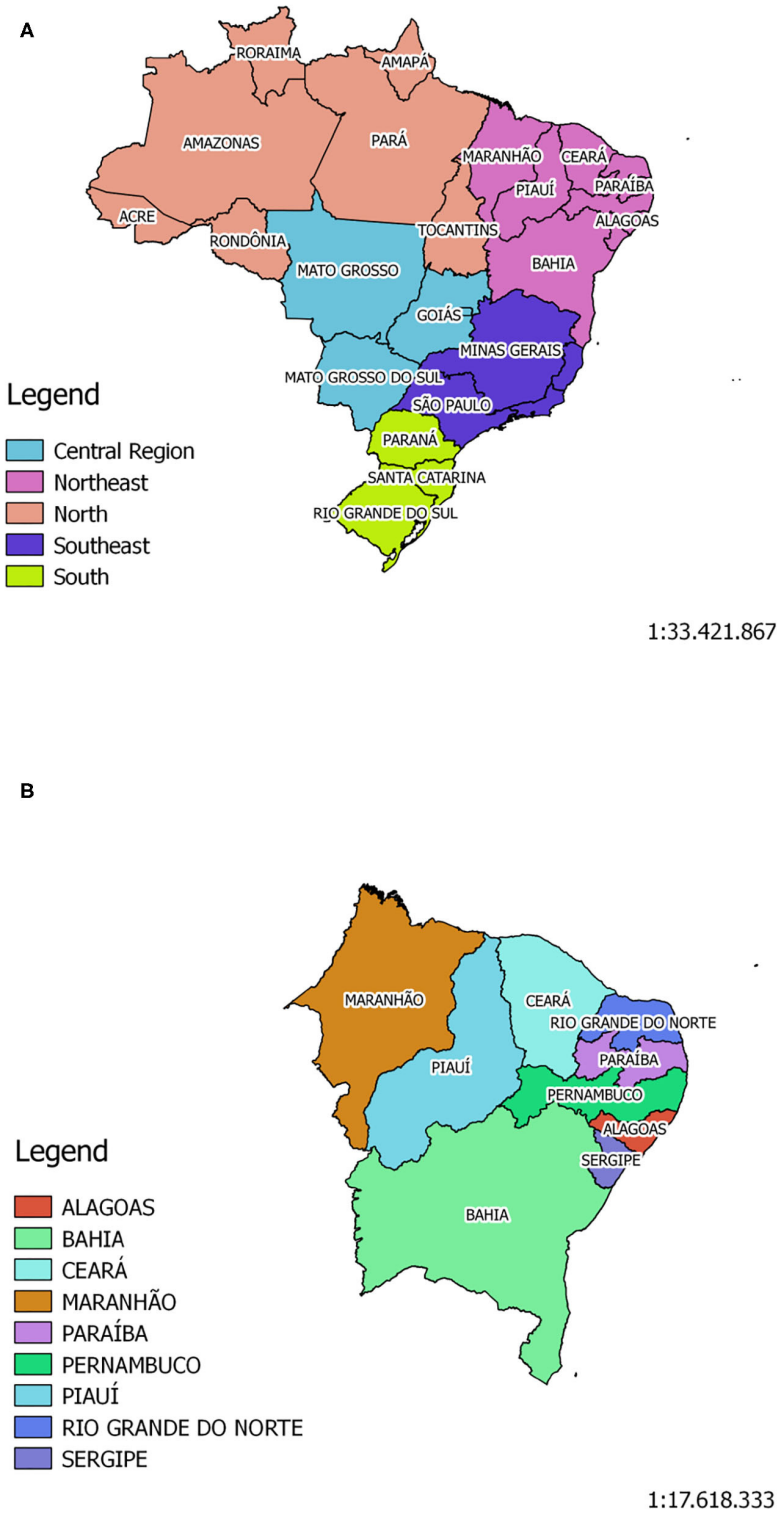
The Brazilian territory is divided in five main regions: North, Northeastern, Midwest, Southern, and South. Brazil has a population of ~210,147,125 million inhabitants, according to Brazilian Institute for Geography and Statistics - IBGE **(A)**. Pernambuco is one of Brazil's 27 federative units, and it is located in the Northeast Region **(B)** of the country. Its population, according to IBGE, is ~9,557,071 million inhabitants.

The areas delimited for this study were Brazil and the State of Pernambuco (Figure 2). Brazil is the largest country located in South America. The country contains 27 federative units and it is divided in five main regions: North, Northeastern, Central Region, Southern and South (Figure 2A). It has a territorial extension of 8,510,820,623 km2, and its population is over 210 million inhabitants. Currently, more than 2 million people were infected with the new corona virus, and ~80,000 people died from the disease (50).

The State of Pernambuco is located in the Northeastern Region of the country (Figure 2B). According to the Brazilian Institute of Geography and Statistics (IBGE), it has a territorial extension of 98,067,881 km2, and its estimated population is around 9.56 million inhabitants. In line with the census taken in 2010, the state has a Human Development Index (HID) of 0.673, being the third highest HID among the northeastern states. To date, Pernambuco has more than 56,000 confirmed cases, and over 18,000 deaths, as reported by the State Health Secretariat (51).

### 3.3. Forecasting Datasets

The data referring to the confirmed cases were obtained through Brasil.io portal. This portal collects daily information regarding confirmed cases and deaths reported in the official bulletins of the State Health Departments. In this work, we collect data for the confirmed cases for both Brazil's and Pernambuco's municipalities, separately. For each territory, we obtained data from the first notification day to June 6, 2020. Then, we calculated the cumulative cases for the two regions for each notification day. The steps described as follows were carried out for both territories, separately.

**Figure 3:**
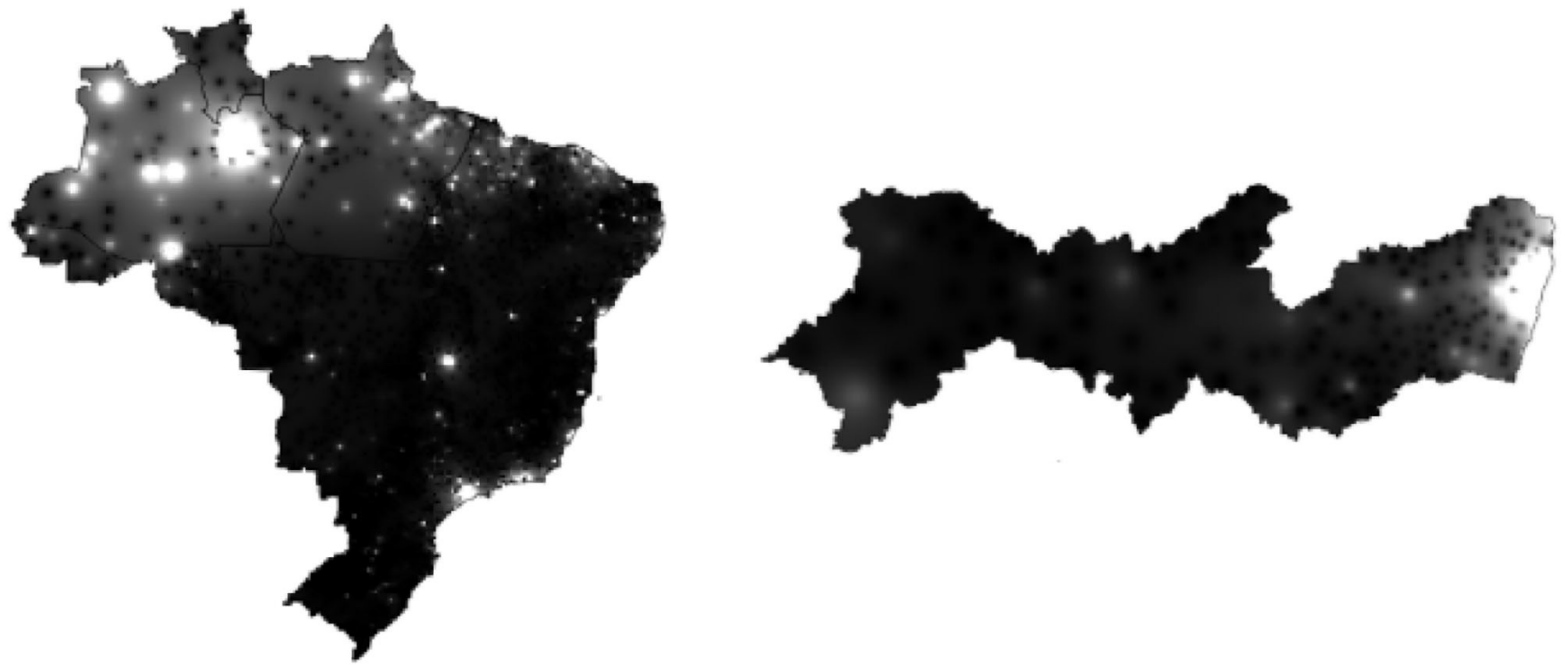
On the right is the Covid-19 accumulated cases's distribution map (gray-level) in the State of Pernambuco on May 5, 2020. On the left is Covid-19 accumulated cases's distribution map in Brazil on May 31, 2020.

**Figure 4:**
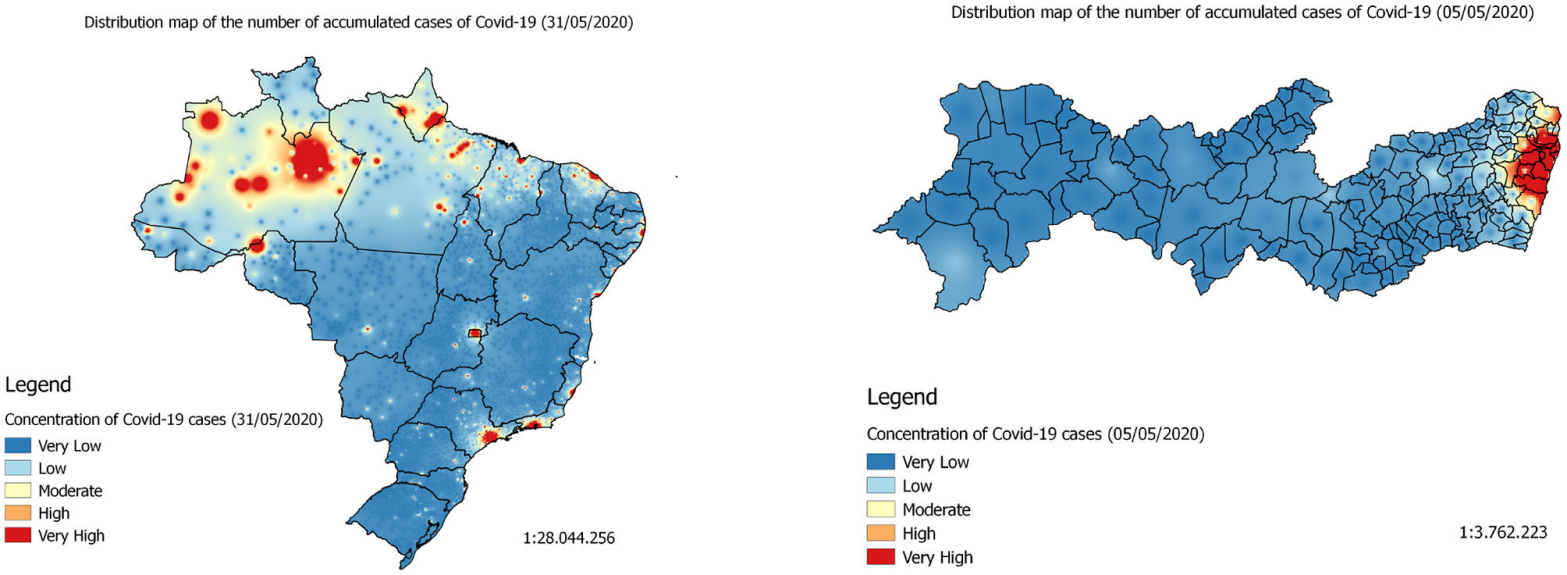
On the right is the Covid-19 accumulated cases's distribution map (heatmap) in the State of Pernambuco on May 5, 2020. On the left is Covid-19 accumulated cases's distribution map in Brazil on May 31, 2020.

In this work, we used a 3-day window to predict the spatial distribution of the following day. Hence, the following step were taken considering four notification days. In this context, firstly we generated point vector layers (.shp) to geolocate the number of accumulated confirmed cases to its respective municipality. Then, for each day of the forecast set, we generated a .shp file using the function *sf_write ()* from the *sf* package in R (52). To estimate the distribution of the disease throughout the territory under study, we generated spatial distribution maps of the accumulated data (Figures 3, 4). The interpolation method chosen was interpolation by the inverse of the distance—with power equal to two—using the *idw ()* function from the *gstat* package in R (53, 54). The function *idw ()* returns a vector for each line of the coordinate tables with the respective interpolated value. The coordinate table is, however, defined by the interpolation grid. Thus, we interpolated the shapefiles generated in the previous step under the interpolation grid of the corresponding territory. For Brazil we used a grid of 90,000 points whereas for Pernambuco we used 15,000. Finally, the vector of the interpolated values were concatenated along with the coordinates information. As a result, we assembled 84 prediction sets for the State of Pernambuco with 15,004 instances, each. For Brazil, on the other hand, we created 100 prediction sets with ~90,000 instances, each. All prediction sets containing five attributes wherein the set output is the pixel value of the accumulated cases's distribution map in the corresponding coordinate.

### 3.4. Regressors

#### 3.4.1. Linear Regression

The linear regression is the simplest method to predict numeric values. In this method, it is assumed that the data has a linear behavior, and that the prediction variable can be represented as a linear combination of the attributes with their pre-determined weights (55). Thus, the general model of linear regression is represented by the Equation (1).

(1) $$\begin{array}{r} y=w_{0}+w_{1}x_{1}+w_{2}x_{2}+\ldots +w_{n}x_{n} \end{array}$$

Where *y* is the prediction variable; *x*_1_, *x*_2_, …, *x*_*n*_, represent the values of the attributes and *w*_0_, *w*_1_, *w*_2_, …, *w*_*n*_ represent the weights of each attribute. The idea of the linear regression algorithm is, then, to find the optimal weights that best represent the problem. One of the ways to find the optimal weights is to minimize the sum of the squared difference between the predicted value and the actual value (55). The sum of the squared difference is calculated by Equation (2).

(2) $$\begin{array}{r} S=\overset{n}{\underset{i=1}{\sum }}{\left[y^{\left(i\right)}-\overset{k}{\underset{j=0}{\sum }}w_{j}x_{j}^{\left(i\right)}\right]}^{2} \end{array}$$

#### 3.4.2. Artificial Neural Networks

Artificial neural networks (ANN), consists in a machine learning technique based on the behavior of the human brain (56). The neural networks consist of smaller units, artificial neurons, which are fundamental to their functioning. The artificial neurons contains the following elements: (1) a set of *synapses* or *connectors*—where a signal *x*_*i*_ at the entrance to the synapse *j* connected to the *k* neuron is multiplied by the synaptic weight *w*_*k,j*_; (2) an *adder* to add the input signals, weighted by the respective neuron synapses; (3) an activation function to limit the output of a neuron (57). Mathematically, an artificial neuron is represented by the Equation (3) and by the Equation (4):

(3) $$\begin{array}{r} u_{k}=\overset{n}{\underset{j=1}{\sum }}w_{k,j}x_{i} \end{array}$$

(4) $$\begin{array}{r} y_{k}=\varphi \left(u_{k}+b_{k}\right), \end{array}$$

wherein *x*_1_, *x*_2_, …, *x*_*n*_ represent the input signals; *w*_*k*,1_, *w*_*k*,2_, …, *w*_*k,n*_ represent the synaptic weights of the input signals *x*_*i*_ for the *k*-th neuron; *b*_*k*_, is the term *bias* and φ is a neuron activation function. In regression applications, the inputs *x*_1_, *x*_2_, …, *x*_*n*_ of the input layer correspond to the forecasting window. For instance, in case of temporal forecasting, the inputs are observed time window of the time series.

**Figure 5:**
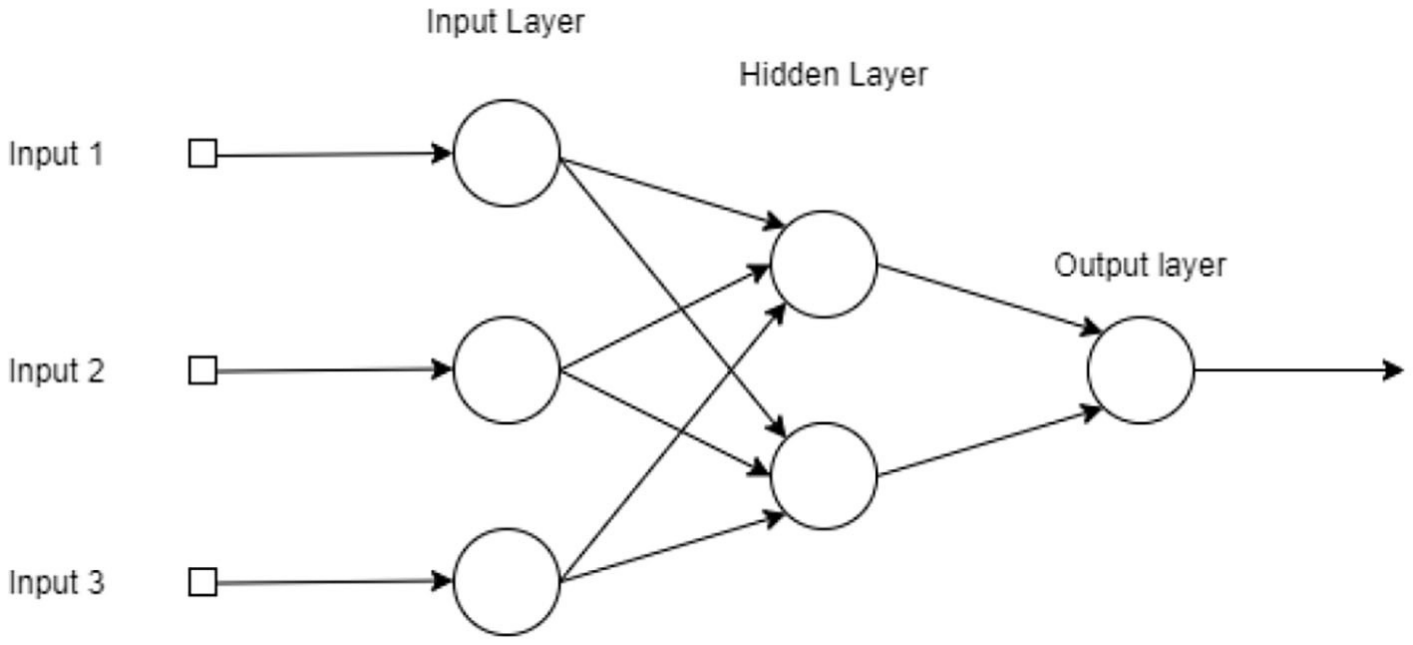
Graphical representation of a Multi-Layer Perceptron with three layers (two hidden layers with three and two neurons, from input to output), a single output and three inputs.

The network architecture used in this work was the multilayer perceptron. In this configuration, the neural network has an input layer, two or more hidden layers, and an output layer (57). In this type of architecture, the neurons input are the output values of the previous layer, as can be seen in the Figure 5. ANNs have also been widely used to predict disease cases. For example, in the prediction of dengue cases in the city of São Paulo, Brazil (58). They were also used to predict dengue outbreaks in the northeastern coast of Yucatán, Mexico, and in San Juan, Puerto Rico (59). Moreover, the ANNs were applied to model cases of infection by *Salmonella* in the state of Mississippi, USA (60).

#### 3.4.3. Support Vector Regression

The support vector regression is a supervised machine learning technique for data analysis and pattern recognition. The idea of the SVR algorithm is to find the best hyperplane defined by Vapnik's ε-insensitivity loss function. When this hyperplane is found, a linear regression is applied to the corresponding hyperplane. In situations where the problem is linearly separable, the best hyperplane is given by the equation:

(5) $$\begin{array}{r} y=w^{T}x+b, \end{array}$$

where $\text{w}={\left(w_{1},w_{2},\ldots ,w_{n}\right)}^{T}$ represents the vector of weights, $\text{x}={\left(x_{1},x_{2},\ldots ,x_{n}\right)}^{T}$ is the vector of the attributes, and *b* is the bias. For problems that are not linearly separable, the data is mapped to a hyperplane in a larger dimension. Thereupon, the algorithm seeks to solve the problem by applying the linear regression of the Equation (5) in the corresponding hyperplane. For non-linearly separable problems, SVR machines use kernel functions, *K* : ℝ × ℝ → ℝ. Then, the SVR output assumes the following expression:

(6) $$\begin{array}{r} y=K\left(w,x\right), \end{array}$$

where the kernel function can be polynomial, sigmoidal, Gaussian, or even assume other mathematical expressions (55, 61, 62).

### 3.5. Regressors Evaluation

The Weka (version 3.8.3) machine learning environment was used to evaluate the regressors. In the case of Brazil, we use its resample tool to evaluate the different architectures. With the resample tool, one can create a new database with random values, but with the same statistical characteristics as the original database. Moreover, the number of instances of the new database can be defined by the user. In this case, we created, for each prediction set in Brazil, a new database with the number of instances equal to 6% of the original set.

Thus, we evaluated four different regression algorithms: Linear Regression, Support Vector Regression (SVR), Multilayer Perceptron, and Random Forest. For the SVR regressor, we performed evaluations with the following configurations: C = 0.1 and linear (or degree 1), 2 and 3-degree polynomial kernels, and RBF (Radial Basis Function) kernel. Regarding MLP, we investigated configurations with 20 and 30 neurons in the single hidden layer. Finally, the random forest algorithm was evaluated for 10, 20, 30, and 40 trees. We investigated each regressor 30 rounds using cross-validation with 10 folds. In order to evaluate the performance of the forecasts, we used the distribution maps from May 25 to 27. The objective is to show the capacity of the proposed spatio-temporal prediction approach to generalize the learning carried out by the regressors considering the 3-day window from 22 to 24 May 2020 to estimate the next 3 days, ie 25, 26, and 27 May 2020. Thus, in this 3-day window approach, not all data from March to June 6, 2020 are used, but only the 3-day window to extrapolate the case numbers for the following days.

### 3.6. Metrics

We selected two metrics to evaluate the models: the correlation coefficient and the Relative Quadratic Error (RMSE percentage). The correlation coefficient is a statistical measure between expected and predicted values. This value varies from −1 to 1. When it approaches 1, it indicates a strong positive correlation. Conversely, when the correlation coefficient is close to −1, it indicates that the variables have a strong negative correlation. Of course, when the correlation coefficient is close to zero, it indicates that there is no correlation between the variables (55). The value of the correlation coefficient serves as the global evaluator for the model—thus, it is possible to obtain a high correlation coefficient as well as at the same time obtain high values for local errors. For this reason, it can not be the only metric for assessing model performance. In order to avoid a superficial evaluation of the regressors, we therefore chose the RMSE as an evaluation metric. The Equation (7) shows the expression of the calculation of the relative quadratic error, where *p*_*i*_ is the predicted value and *a*_*i*_ is the actual value, for *i* = 1, 2, …, *n*.

(7) $$\begin{array}{r} \text{RMSE}\left(\%\right)=\sqrt{\frac{\sum_{i=1}^{n}{\left(p_{i}-a_{i}\right)}^{2}}{\sum_{i=1}^{n}a_{i}^{2}}}\times 100\%. \end{array}$$

## 4. Results

### 4.1. Regression Experiments Results

The models were evaluated according to the metrics described in the 3.6 section. The RMSE (%) was used as a metric of local quality whereas the correlation coefficient was used as a metric of global quality. In this work, we consider a high correlation coefficient to be above 0.9 and a low RMSE (%) to be below 5%. Tables 1, 2 show the evaluation metrics of the results for the models using the regressors mentioned in section 3.5.

**Table 1:** Results of the performance of the linear regression algorithms, the multilayer perceptron (MLP), and the support vector regressor (SVR) for Brazil's data set.

		**Correlation coefficient**		**RMSE (%)**		**Training time (s)**	
**Regression method**	**Configuration**	**Average**	**Standard deviation**	**Average**	**Standard deviation**	**Average**	**Standard deviation**
Linear regression	–	0.9826	0.143	11.42	22.94	0.003	0.006
	10 trees	0.9737	0.072	22.35	24.08	0.06	0.01
	20 trees	0.9752	0.070	21.59	23.03	0.20	0.04
	30 trees	0.9756	0.069	21.32	22.68	0.29	0.06
Random forest	40 trees	0.9759	0.068	21.17	22.33	0.22	0.04
	20 neurons	0.9948	0.006	11.29	7.12	13.91	0.19
MLP	30 neurons	0.9867	0.011	17.9	4.12	6.48	0.30
	Polynomial kernel, *p* = 1	0.9670	0.109	18.39	24.08	3.82	2.14
	Polynomial kernel, *p* = 2	0.9818	0.006	31.70	10.62	7.99	10.57
	Polynomial kernel, *p* = 3	0.9623	0.004	45.15	17.35	7.55	9.45
SVR	RBF kernel	0.5341	0.375	87.19	14.94	39.97	56.17

*For each regressor, we calculated the correlation coefficient, the relative square error [RMSE (%)], and the training time*.

**Table 2:** Results of the performance of the linear regression algorithms, the multilayer perceptron (MLP), and the support vector regressor (SVR) for Pernambuco's data set.

		**Correlation coefficient**		**RMSE (%)**		**Training time(s)**	
**Regression method**	**Configuration**	**Average**	**Standard deviation**	**Average**	**Standard deviation**	**Average**	**Standard deviation**
Linear regression	–	0.9991	0.006	1.92	3.81	0.02	0.01
	10 trees	0.9983	0.003	6.14	4.14	0.27	0.05
	20 trees	0.9985	0.003	5.78	4.03	0.52	0.09
	30 trees	0.9985	0.003	5.65	4.01	0.78	0.11
Random forest	40 trees	0.9985	0.003	5.57	3.99	1.21	0.14
	20 neurons	0.9990	0.005	4.07	4.95	45.82	1.56
MLP	30 neurons	0.9991	0.005	3.81	4.95	62.69	3.60
	Polynomial kernel, *p* = 1	0.9988	0.006	3.28	4.05	18.61	22.15
	Polynomial kernel, *p* = 2	0.9964	0.011	6.48	5.85	82.79	34.22
	Polynomial kernel, *p* = 3	0.9989	0.001	4.48	1.99	89.64	8.34
SVR	RBF kernel	0.9387	0.064	76.86	13.82	112.78	122.81

*For each regressor, we calculated the correlation coefficient, the relative square error [RMSE (%)], and the training time*.

Table 1 shows the findings of the experiments with the database for Brazil's territory. It shows the average and standard deviation of the evaluation metrics for each regressor investigated. Thus, for linear regression, we obtained, on average, a correlation coefficient of 0.9826, with a standard deviation of 0.143. As for the RMSE (%), we obtained an average of 11.42%, with a standard deviation of 22.94%. Although this method obtained a high correlation coefficient, its error was higher than the value we established for this work. The findings in Table 1 also show that for the random forest method, the average of the models' correlation coefficients ranged from 0.9737 to 0.9759 (with standard deviations ranging from 0.072 to 0.068). Similarly, the values of the RMSE (%) did not show a large variation among the evaluated configurations. The configuration with the lowest error was the configuration with 40 trees where the average of RMSE (%) for the generated models was 21.17%. The experiments with the multilayer perceptron showed an error of 11.29 and 17.90% for configurations with 20 and 30 neurons, respectively. However, both achieved a high correlation coefficient, that is, above 0.9.

The models generated by the support vector machines did not achieved a good performance, as well. Among the configurations evaluated, the configuration with the degree-1 polynomial kernel showed the best results. For this SVR configuration, the average of the correlation coefficients was 0.9670 and RMSE (%) 18.39. In contrast, the experiments with the RBF kernel presented the worst performance, with a correlation coefficient of 0.5341 and a very high RMSE (%), reaching around 87%.

Table 2 shows the results of the regressors's performance for the distribution maps of the accumulated cases in the State of Pernambuco. The linear regression algorithm performed with correlation coefficient higher than 0.9, while the RMSE (%) obtained was 1.92%. The random forest algorithm, in turn, did not show a great variation between the different configurations tested. As for the correlation coefficient, all configurations obtained a very high value, above 0.9. On the other hand, the errors varied between 5.57 and 6.14%, where the lowest value corresponds to the configuration with 40 trees, and the highest value corresponds to the configuration with 10 trees. As for the two neural network configurations tested, both showed a good performance. The means of the correlation coefficients were 0.9990 and 0.9991 for configurations with 20 and 30 neurons, respectively. In addition, the RMSE (%) was around 4%, which is slightly below the 5% established in the section 3.6.

As previously described, we generated three regular-grid distributions from irregular daily distributions by using IDW interpolation for each spatio-temporal forecast. Since Brazil is a continental country with a considerable diversity of natural biomass, federative units like Amazonas and Pará are composed by too large municipalities, many of them with reduced population density. Thus, the centroids of the municipalities tend to be too far from each other, generating a relatively sparse region of the irregular grid. In this case, the IDW interpolation method tends to generate estimatives with high local errors. Therefore, for the whole country of Brazil, RMSE (%) measures tend to be higher than expected (cf. Table 1), showing a considerably different behavior than the same metric for the State of Pernambuco (cf. Table 2).

Finally, among the evaluated SVR configurations, the one with the best performance was the polynomial kernel with degree 1. The models generated by this regression method had a correlation coefficient on average of 0.9988 and an error of 3.28% (see Table 3). Like the results from Brazil, the RBF kernel showed the worst performance among this set of algorithms. The RMSE (%) obtained was ~77% despite having obtained a high correlation coefficient.

**Table 3:** Results of the validation for the models created using linear regression and SVR, RBF kernel, for Brazil and Pernambuco.

	**Regression method**	**Prediction date**	**Correlation coefficient**	**RMSE (%)**
PERNAMBUCO	Linear regression	25/05/2020	0.999994	1.32
		26/05/2020	0.999990	3.27
		27/05/2020	0.999970	3.84
BRAZIL	Linear regression	25/05/2020	0.998447	5.68
		26/05/2020	0.998840	5.03
		27/05/2020	0.978811	20.86
PERNAMBUCO	SVR, kernel = RBF	25/05/2020	0.949280	86.55
		26/05/2020	0.869430	99.33
		27/05/2020	0.511389	101.2
BRAZIL	SVR, kernel = RBF	25/05/2020	0.993423	42.15
		26/05/2020	0.951116	71.23
		27/05/2020	0.751036	87.91

*We forecasted the distribution of accumulated cases of Covid-19 between May 25 and 27, 2020*.

### 4.2. Web Application

**Figure 6:**
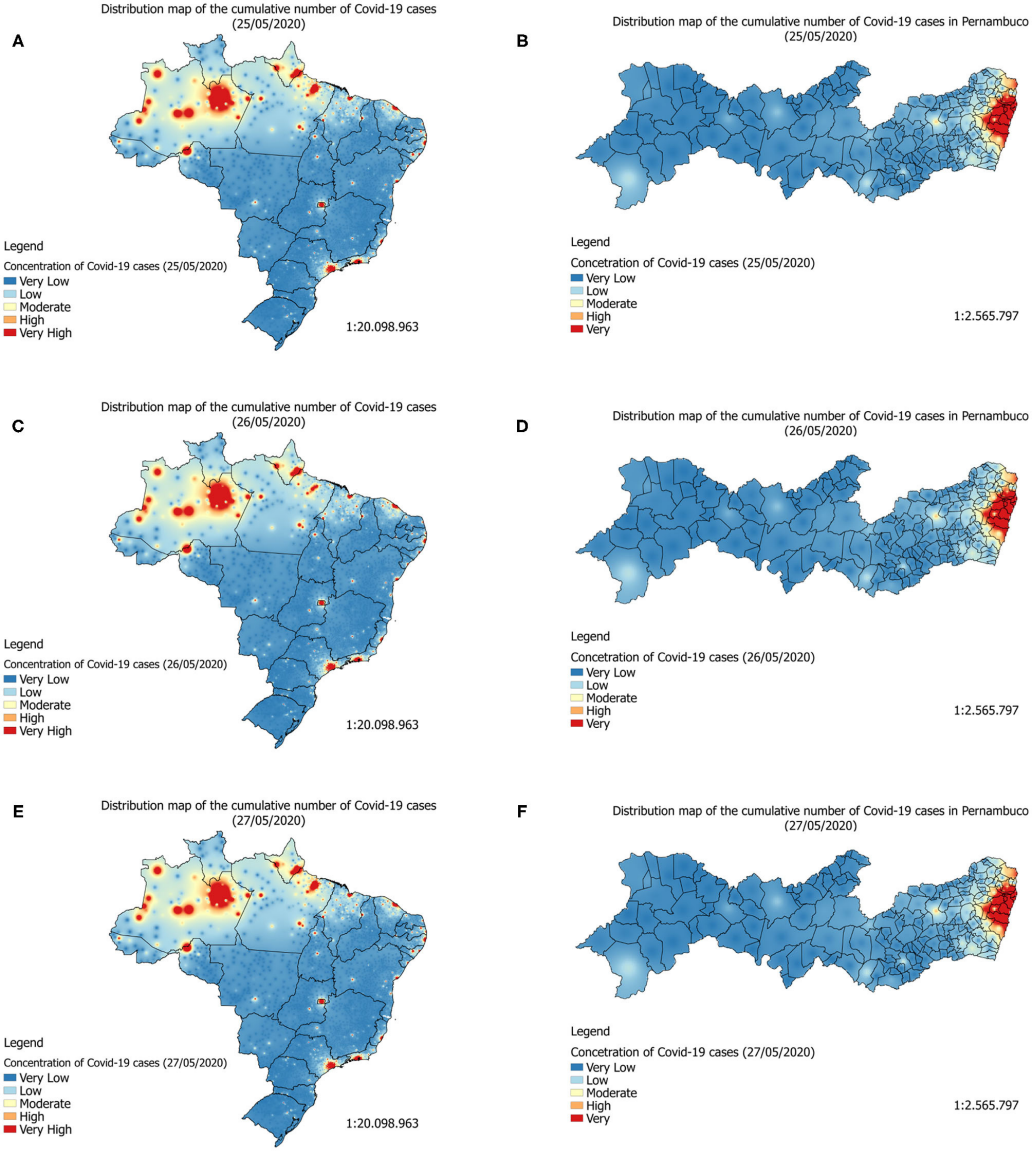
Forecast maps (heatmaps) of Covid-19's distribution in Brazil between May 25 and 27, 2020 **(A–C)**. Forecast maps of Covid-19's distribution in Pernambuco between May 25 and 27, 2020 **(D–F)**.

The developed application can be accessed through the link (https://www.cin.ufpe.br/~covidsgis). On the home screen, the user can visualize the Covid-19 monitoring (Figure 6A). In this section, the number of accumulated and daily cases of Covid-19 in each state are available. In the lower right corner of the home screen, on the “Change map” option, the user can select the spatio-temporal prediction maps (Figure 6B). In these maps, it is possible to visualize the regions of Brazil which present a high, medium and low density of the accumulated cases of Covid-19 (Figure 6C).

In the lower right corner of the home screen, on the “More information” option, the user is directed to the graphs screen. In this screen, the graphs of the temporal forecast of the accumulated cases and the accumulated deaths by Covid-19 are presented for each of the 27 federative units in Brazil (Figure 6D). In addition to the forecast graphs, it is possible to select the graphs of accumulated cases, daily cases, accumulated deaths and daily deaths (Figures 6E,F). These graphs can be viewed both for the states of Brazil and for the Brazilian cities. In this screen, the users can also select personalized dashboards for each of the 27 federative units in Brazil to visualize information about Covid-19 at municipality level (Figures 6G,H).

## 5. Discussion

In this study, we evaluated the prediction of the maps of distribution of the accumulated cases of Covid-19 for the territory of Brazil and for the State of Pernambuco. We used machine learning techniques and the models generated were evaluated for the period of 25 and 27 May. In general, for the Brazil's territory, the regressors that showed the best performances were linear regression, and MLP with 20 neurons in the hidden layer. On the other hand, the SVR with RBF kernel showed a bad performance. For this regressor the errors reached more than 90%, making it not suitable for solving this problem.

Our findings also showed that the linear regression for the spatial prediction of the accumulated cases in the State of Pernambuco presented a very good performance. This may indicate that the spread of Covid-19 over the territory of Pernambuco has a linear behavior. As the results from Brazil, the SVR with an RBF kernel showed the worst performance among all the regressors evaluated. For this SVR configuration, the RMSE (%) reached 87%.

**Figure 7:**
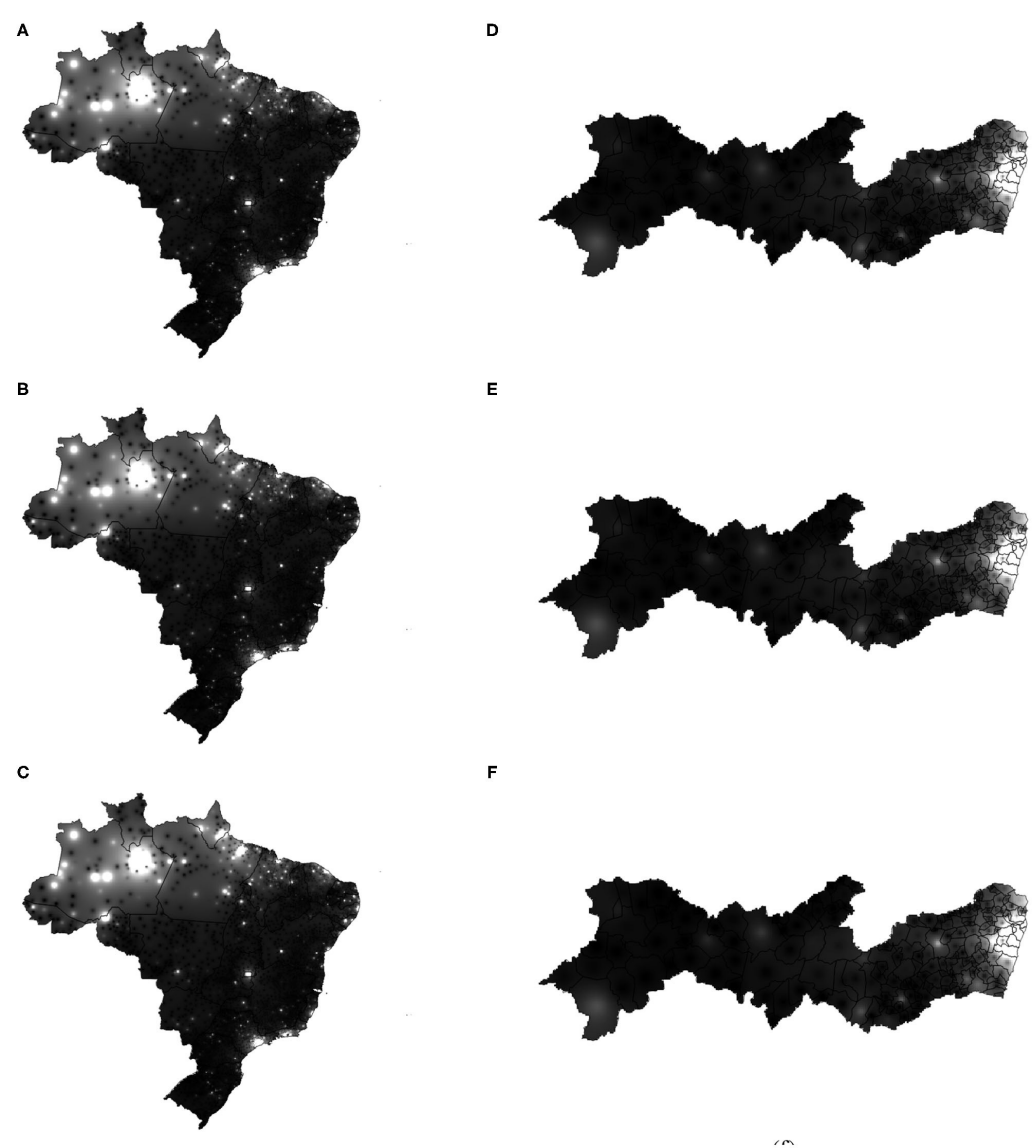
Forecast maps (gray-level) of Covid-19's distribution in Brazil between May 25 and 27, 2020 **(A,C,E)**. Forecast maps of Covid-19's distribution in Pernambuco between May 25 and 27, 2020 **(B,D,F)**.

**Figure 8:**
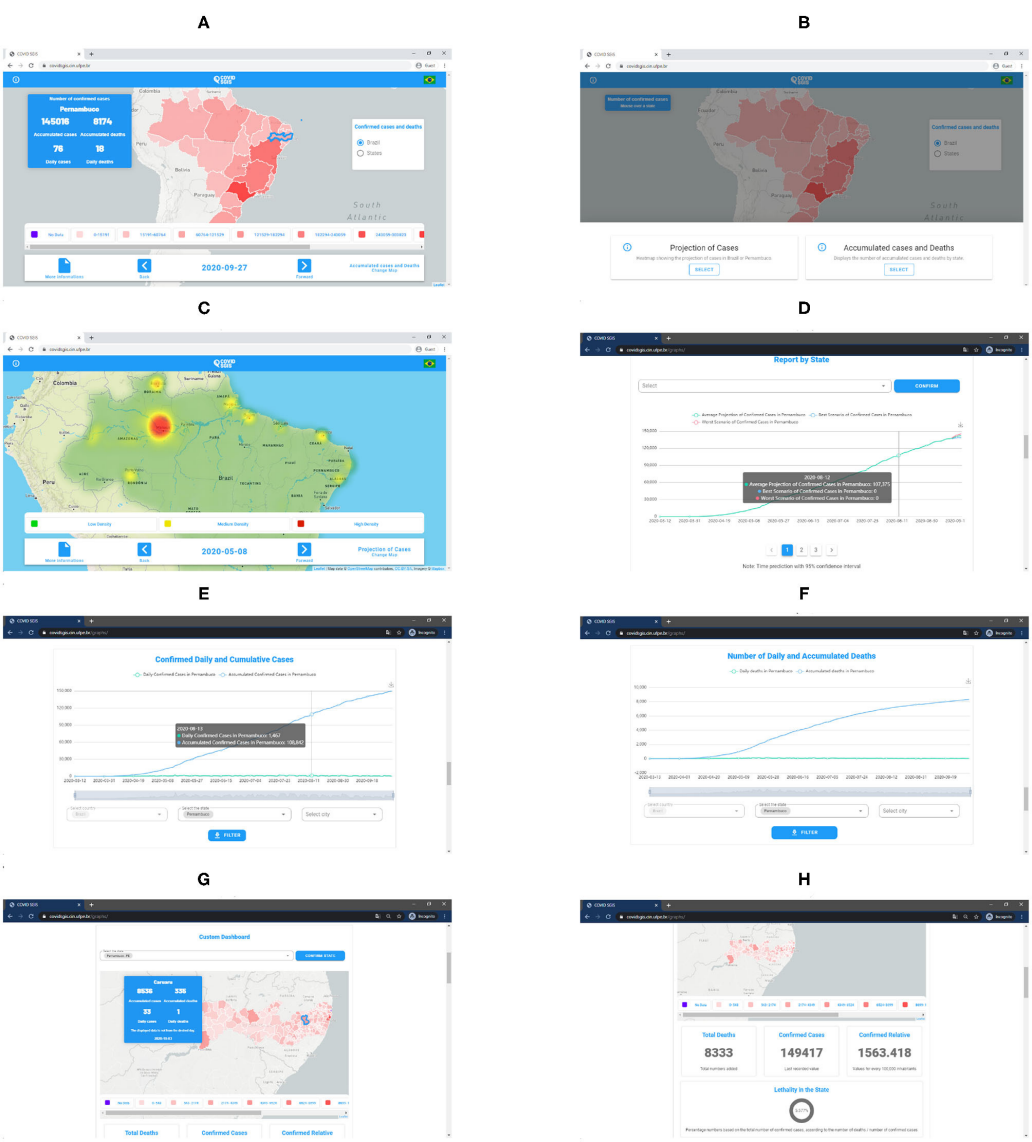
**(A)** COVID-SGIS home screen. **(B)** Map selection screen, wherein it is possible to visualize the cumulative cases' map and the forecasted distribution maps. **(C)** Screen of the prediction of the distribution maps of the cumulative cases of Covid-19. **(D)** Accumulated cases of Covid-19 forecast graph with ARIMA. The green line represents the ARIMA forecast, whereas the red and the blue lines represent the worst and the best scenario. Screen of the graphs for accumulated and daily confirmed cases **(E)**, and accumulated and confirmed deaths **(F)**. Screens of the personalized dashboard **(G,H)**. The personalized dashboard gives detailed information—regarding the cumulative confirmed cases and daily cases, and cumulative deaths and daily deaths—for each state at a municipality level).

By analyzing the prediction maps of Figures 7A,C,E (heatmaps) and Figures 8A–C (heatmaps), it is observed that, in Brazil, the region that has the highest concentration of cases in Covid-19 is the North Region. For this region, according to the maps, the states of Amazonas, Pará, and Amapá. In these states, much of the territory has a high concentration of cases. In the Northeast Region, the states with the highest concentrations of cases are the states of Maranhão and Ceará, followed by the State of Pernambuco (see Figures 7B,D,F, 8D–F). In the Southeast Region, the states of São Paulo and Rio de Janeiro have the highest concentrations of the accumulated cases of Covid-19 in their territories. It is observed that, although São Paulo has the largest number of confirmed cases of the disease, the state of Amazonas is the one that is suffering the most from the pandemic. The State of Amazonas is a region very sensitive to Covid-19, due to the large population of indigenous people and their descendants, who are part of the group at risk of the disease. The large concentration of cases in this state may be linked to the absence of measures to encourage social isolation, closing the borders of the state, which would be essential for the disease to reach the interior of the state (63).

In the State of Pernambuco, the cases are concentrated in the extreme east of the state, mainly in the state capital, the City of Recife. Gradually the disease spread through the interior of the state, reaching mainly the cities of Caruaru and Petrolina, which are cities interconnected by the main highways that cut the state (64).

## 6. Conclusion

Covid-19 has been a major public health challenge worldwide. To date, more than 235 countries have been affected by the pandemic (65). Brazil is the second country with the highest number of confirmed cases of the disease, behind only the United States (65). With that in mind, it is important to forecast the spatial distribution of the cumulative cases of Covid-19, so that public health managers can evaluate better strategies to slow the progress of the disease and prevent the appearance of a second wave.

The use of machine learning proved to be very effective in forecasting the spatial distribution of the cumulative cases of Covid-19. For both territories, the linear regression showed the best performance whereas the SVR, kernel RBF presented the worst results. As our qualitative results showed, in Brazil, the states with the highest concentration of cases were the states of the North Region (Amazonas, Pará, and Amapá). On the other hand, in Pernambuco, the far east of the state has the highest concentration Covid-19's cases. The disease gradually spread to the interior of the state. In this study, we do not consider the climatic variables to generate the models, nor the sociodemographic factors that may influence the dynamics of the disease.

Finally, the approach using spatio-temporal analysis provided a broader assessment of those in the regions where the accumulated confirmed cases of Covid-19 are concentrated. From the qualitative results it was possible to differentiate in the heat maps the regions with the highest concentration of cases from the regions with low concentration and the regions that are in the transition range. This type of approach proves to be quite relevant in supporting health managers and epidemiologists regarding the planning of disease prevention actions.

## Data Availability Statement

Publicly available datasets were analyzed in this study. This data can be found at: https://brasil.io/home/, https://github.com/Biomedical-Computing-UFPE/Covid-SGIS.

## Author Contributions

CS: responsible for the design of forecasting models using machine learning. CL: responsible for the design of forecasting models using machine learning and models' implementation in R code. AS, MS, JG, and VB: responsible for models' implementation in R code. ES, GM, LA, LAJ, and SS: frontend and back-end developers. AM, PK, WS, and AF: associate researchers and supervisors. All authors contributed to the article and approved the submitted version.

## Conflict of Interest

The authors declare that the research was conducted in the absence of any commercial or financial relationships that could be construed as a potential conflict of interest.
